# Expression of E-cadherin and KRAS mutation may serve as biomarkers of cetuximab-based therapy in metastatic colorectal cancer

**DOI:** 10.3892/ol.2013.1187

**Published:** 2013-02-08

**Authors:** KENTARO NAKAMOTO, HISASHI NAGAHARA, KIYOSHI MAEDA, EIJI NODA, TORU INOUE, MASAKAZU YASHIRO, YUKIO NISHIGUCHI, MASAICHI OHIRA, KOSEI HIRAKAWA

**Affiliations:** 1 Department of Surgical Oncology, Osaka City University Graduate School of Medicine, Osaka 545-8585, Japan; 2 Department of Gastroenterological Surgery, Osaka City General Hospital, Osaka 545-8585, Japan

**Keywords:** E-cadherin, KRAS mutation, mCRC, cetuximab, biomarker

## Abstract

Cetuximab (Cmab), a chimeric monoclonal antibody for targeting the epidermal growth factor receptor, has become one of the standard treatments for metastatic colorectal cancer (mCRC). However, only a small proportion of patients respond to Cmab, and it has been reported that *KRAS* mutation is a negative biomarker of response to Cmab therapy. The aim of this study was to detect additional biomarkers of response to Cmab therapy in patients with mCRC. We evaluated the effects of Cmab therapy in 36 patients with mCRC according to the Response Evaluation Criteria in Solid Tumors, and classified patients who achieved complete response, partial response or stable disease as responders, and patients who achieved progressive disease as non-responders. We retrospectively examined the difference between the two groups using *KRAS* analysis and immunohistochemistry to determine the expression of E-cadherin, p53 and Ki67. Nineteen patients were responders, while 17 patients were non-responders. *KRAS* status and expression of E-cadherin were significantly correlated with the effect of Cmab therapy. Moreover, the expression of E-cadherin was significantly correlated with the effect of Cmab therapy in *KRAS* wild-type patients. In *KRAS* mutant-type patients, the expression of E-cadherin did not significantly correlate with the effect of Cmab therapy, but all responders with *KRAS* mutant-type tumors expressed E-cadherin. Our results indicate that the expression of E-cadherin detected by immunohistochemistry may be a positive predictor of Cmab-based therapy in mCRC, and that a combination of E-cadherin immunohistochemistry and *KRAS* analysis may be a more sensitive biomarker than *KRAS* analysis alone.

## Introduction

Colorectal cancer (CRC) is one of the most common types of cancer in the world. Despite advances in chemotherapeutic agents, the prognosis for patients with metastatic CRC (mCRC) remains poor (1). Cetuximab (Cmab) is a chimeric monoclonal antibody (moAb) for epidermal growth factor receptor (EGFR), and has been shown to be effective for mCRC in combination with chemotherapy or as a single agent (2–6). EGFR is expressed in various malignancies, including CRC (7). EGFR activation plays an important role in growth and progression, involving proliferation, angiogenesis, invasion and metastasis (8). Cmab binds to the extracellular domain of EGFR and inhibits downstream signal transduction (9). However, only 10–20% of patients with mCRC respond to Cmab (3). The identification of biomarkers of response to Cmab for mCRC is important in the selection of mCRC patients who should be administered Cmab to avoid unnecessary toxicities and ineffective, expensive therapy. Analysis of clinical trials for mCRC indicates that *KRAS* mutation is a negative predictor of Cmab-based therapies (10–14). *KRAS* belongs to the oncogene family of genes and is activated by EGFR which binds to a ligand (8). *KRAS* mutation continuously activates downstream RAS-RAF-MAPK pathways whether EGFR is activated or blocked by the antibody (8). Although *KRAS* mutation may be considered a highly specific negative biomarker of response, it is also poorly sensitive (15). The identification of additional biomarkers is necessary to improve sensitivity. EGFR copy number (16–18), the levels of expression of amphiregulin and epiregulin (19), FCGR2A and FCGR3A polymorphisms (20), BRAF mutation, PIK3CA mutation and PTEN inactivation (18,21–26) have been reported to be associated with response to Cmab, but at present, these markers cannot be used to select patients who are eligible for Cmab treatment.

A recent study revealed that *p53* mutations are predictive of Cmab sensitivity (27). Another study reported that Ki67 expression is downregulated following Cmab-based neoadjuvant chemoradiotherapy in rectal cancer (28). Moreover, it has been reported that expression of E-cadherin is a marker of response to Cmab *in vitro*(29). In the present study, we examined the expression of p53, Ki67 and E-cadherin together with *KRAS* status and assessed their predictive value as biomarkers of response to Cmab in mCRC.

## Materials and methods

### Patients and tissue samples

We assessed 36 mCRC patients treated with Cmab-based therapy, who had tumor tissues available for molecular analysis. Tumor response was evaluated according to the Response Evaluation Criteria in Solid Tumors (RECIST). Patient tumor response was classified as complete response (CR), partial response (PR), stable disease (SD) or progressive disease (PD). Patients who achieved PR or CR or SD were considered responders (controlled disease; CD). Patients who achieved PD were considered non*-*responders. Follow-up was performed on a clinical basis and CT scan until disease progression, mortality or the last follow-up point at which data were monitored. The study was conducted in accordance with the Helsinki Declaration and was approved by the Ethics Committee of Osaka City University, Osaka, Japan. Informed consent was obtained from all patients or guardians.

### DNA extraction

DNA was extracted from tissue sections fixed in 10% buffered formalin and embedded in paraffin. An adjacent section stained with hematoxylin and eosin was used as a guide in the selection of areas for microdissection under a dissecting microscope, using a sterile scalpel blade. Genomic DNA was extracted from the paraffin-embedded tissue using Proteinase K (Gibco-BRL, Gaithersburg, MD, USA).

### Dot-blot hybridization

The DNA was amplified using a heminested PCR protocol as previously described (30). PCR amplification of exon 2 of a *KRAS*-containing codons 12 and 13 was first performed using the following primers: forward, 5’-CGTCCACAAAATGATTCTGAATTAGCTGTATC-3’ and reverse, 5’-CCTTATGTGTGACATGTTCTAATATAGT CAC-3’. Thirty-five cycles (92°C for 30 sec and 67°C for 30 sec) were performed, followed by a 10-min extension at 72°C. Initial PCR products were diluted and further amplified using a new forward primer, 5’-AGGCCTGCTGAAAATGAC-3’, and the same reverse primer described above. Thirty-five cycles (92°C for 25 sec, 55°C for 25 sec and 72°C for 25 sec) were performed, followed by a 10-min extension at 72°C. The 104-bp amplicons were then dot-blotted onto nylon filters (Hybond-N; Amersham, Buckinghamshire, UK) and hybridized with radiolabeled oligomer primers representing all possible mutations at codon 12 and the GAC mutation of codon 13. Direct sequencing was performed to confirm the presence of KRAS mutations at codons 12 and 13, which were detected by dot-blot hybridization.

### Immunohistochemical study

All tissues were fixed in 10% formalin immediately after surgical resection or biopsy and embedded in paraffin. The slides were deparaffinized and heated for 10 min at 105°C by autoclave in Target Retrieval Solution (Dako, Carpinteria, CA, USA). Sections were then incubated with 3% hydrogen peroxide to block endogenous peroxidase activity. Thereafter, sections were incubated in 10% normal goat or rabbit serum to reduce non-specific antibody binding. Primary monoclonal antibodies were directed against p53 (DO7, dilution 1:50; Dako), Ki67 (MIB-1, dilution 1:50; Dako) and E-cadherin (clone NCH-38, dilution 1:200; Dako). Tissue sections were incubated with each antibody overnight at 4°C. After washing in phosphate-buffered saline (PBS), tissues were incubated with horseradish peroxidase-conjugated anti-rabbit or anti-mouse Ig polymer as a secondary antibody (Envision kit; Dako) for 30 min at room temperature, according to the manufacturer’s instructions. The slides were treated with streptavidin-peroxidase reagent and incubated in PBS and diaminobenzidine and 1% hydrogen peroxide v/v, followed by counterstaining with Mayer’s hematoxylin. Positive and negative controls for each marker were used according to the manufacturer’s instructions (Dako). The immunostained slides were independently examined and scored by two investigators. Immunohistochemical scoring was performed in a blind manner. p53 expression was semi-quantitatively analyzed according to the percentage of cells showing nuclear positivity: 0, 0 to 10%; 1+, >10 to 25%; 2+, >25% to 50%; 3+, >50%. According to previous studies, p53 expression was considered positive when scores were >1, and negative when scores were 0 (31–34). For the tissue evaluation of Ki67, each slide was scored based on the percentage of positively stained malignant nuclei. According to the recommended classification in previous studies, the cut-off Ki67 positivity was >40% positive tumor cells with nuclear staining (32,33,35). E-cadherin antibody stained the membrane intensely and the cytoplasm of cancer cells weakly. E-cadherin expression was semi-quantitatively analyzed according to the percentage of cells showing membrane positivity: 0, 0%; 1+, >0 to 25%; 2+, >25 to 50%; 3+, >50%. According to previous studies, E-cadherin expression was considered positive when scores were >1 and negative when scores were 0 (36,37). A case with cytoplasmic staining only was determined as E-cadherin-negative.

### Statistical analysis

Statistical analysis was performed using SPSS 13.0 statistical software (SPSS Inc., Chicago, IL, USA). We examined the association between the biological parameter status and treatment response using Chi-square analysis. We also estimated odds ratios (ORs) using logistic regression analysis. All P-values were 2-sided and P<0.05 was considered to indicate a statistically significant difference. Cut-off values for different biomarkers included in this study were selected before statistical analysis.

## Results

A total of 36 mCRC patients treated with Cmab-based therapy, including 24 males and 12 females with a mean age of 62.2 years (range, 29–79) were included in this study (Table I). Twenty-seven patients received Cmab-based therapy as third line therapy, 8 patients received it as second line and only one patient received it as first line therapy. With regard to concurrent chemotherapy, 17 patients received Cmab with irinotecan, 18 received Cmab alone and one patient received Cmab with mFOLFOX6. Response to Cmab therapy demonstrated that 19 (53%) patients had a CD (8 PR and 11 SD), while 17 (47%) were in PD.

### KRAS status analysis

Table II shows the results of *KRAS* status analysis. Results of dot-blot hybridization were equivalent to that of direct sequencing. *KRAS* was mutated in 12 (33%) of 36 tumors. Ten (83%) of 12 tumors had *KRAS* mutation in codon 12, while 2 (17%) of 12 had mutations in codon 13.

### Expression of p53, Ki67 and E-cadherin demonstrated by immunohistochemistry (IHC)

**Figure 1:**
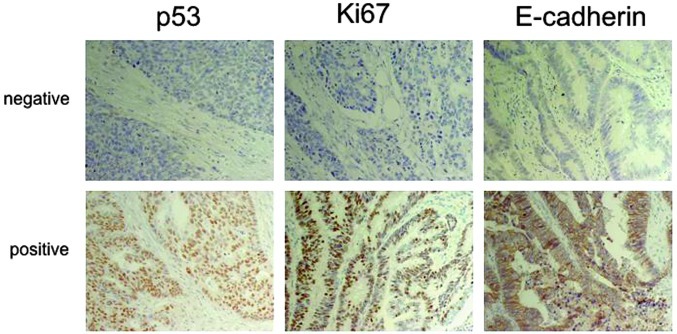
Immunohistochemical expression of p53, Ki67 and E-cadherin. (magnification, ×200). Upper panel, negative cases; lower panel, positive cases. p53 and Ki67 expression was observed in the nuclei, and E-cadherin expression was detected in the cell membrane.

Fig. 1 shows the expression of p53, Ki67 and E-cadherin. p53, Ki67 and E-cadherin were positive in 29 (81%), 21 (58%), and 22 (61%) of 36 tumors, respectively.

### Correlation between response to Cmab and KRAS status, and expression of p53, Ki67 and E-cadherin

Table III shows the response to treatment with Cmab according to *KRAS* status, and expression of p53, Ki67 and E-cadherin. Sixteen (67%) of 24 patients with KRAS wild-type tumors were found in responders compared with 3 (25%) of 12 patients with *KRAS* mutant-type tumors in responders. Seventeen (77%) of 22 patients with E-cadherin-positive tumors were found in responders, compared with 2 (14%) of 14 patients with E-cadherin-negative tumors found in responders. *KRAS* status and expression of E-cadherin were significantly associated with response to Cmab treatment (P=0.033 and P<0.001, respectively). Expression of p53 and Ki67 were not associated with response to Cmab treatment (P=0.219 and P=1.000, respectively).

### Expression of E-cadherin in KRAS wild-type patients

E-cadherin was positive in 14 (58%) of 24 *KRAS* wild-type tumors. Fourteen (93%) of 15 patients with E-cadherin-positive tumors were found in responders compared with 2 (22%) of 9 patients with E-cadherin-negative tumors found in responders. Expression of E-cadherin was significantly associated with response to Cmab treatment in *KRAS* wild-type patients (P=0.001; Table IV).

### Expression of E-cadherin in KRAS mutant-type patients

E-cadherin was positive in 7 (58%) of 12 *KRAS* mutant-type tumors. Expression of E-cadherin was not significantly associated with response to Cmab treatment in *KRAS* mutant-type patients (P=0.205). However, all 3 responders with *KRAS* mutant-type tumors expressed E-cadherin (Table IV).

### Univariate and multivariate models

In the univariate analysis, which included age (<62 vs. ≥62 years), gender (male vs. female), site of tumors (colon vs. rectum), concurrent chemotherapy (yes vs. no), *KRAS* status (wild-type vs. mutant), expression of p53, Ki67 and E-cadherin with IHC (positive vs. negative), only *KRAS* status and expression of E-cadherin demonstrated a significant association with response to treatment with Cmab. In the multivariate analysis, *KRAS* status and E-cadherin IHC significantly affected the efficacy of Cmab-based therapy (*KRAS*: OR, 20.83; 95% CI, 1.80–241.18; P=0.015; E-cadherin: OR, 54.91; 95%CI, 4.53–664.89; P=0.002; Table V). No evidence of interaction between *KRAS* status and expression of E-cadherin was detected.

## Discussion

E-cadherin is a calcium-regulated homophilic cell-cell adhesion molecule. Previous studies have reported that E-cadherin regulates not only cell-cell adhesion, but also intracellular signaling cascades, including the Akt and MAPK pathways (38,39). It has been revealed that E-cadherin coexists with EGFR in a complex and the extracellular domain of E-cadherin regulates the ability of EGFR to respond to its ligand (40). Furthermore, it has been reported that there is a significant correlation between expression of E-cadherin and sensitivity to the EGFR tyrosine kinase inhibitor, gefitinib, in non-small cell lung cancer cell lines (41), and that the most gefitinib-sensitive cell lines have higher levels of EGFR activation (42). In addition, loss of E-cadherin has been demonstrated to be a marker of poor response to the antiproliferative effect of Cmab in a panel of urothelial carcinoma cell lines (29). These findings suggest that cells expressing E-cadherin increase dependence on EGFR for cell growth and survival and that cells lacking E-cadherin have developed other activating mechanisms that bypass EGFR signaling for cell growth and survival, and then acquire resistance to EGFR inhibition. Based on these findings, we hypothesized that the expression of E-cadherin, detected with IHC, may be a biomarker of response to Cmab in mCRC. This is the first study to investigate the correlation between the expression of E-cadherin demonstrated by IHC and the effect of Cmab in mCRC clinical specimen. In our experience, expression of E-cadherin correlated with a higher controlled disease rate in mCRC treated with Cmab.

Our results are consistent with the knowledge that KRAS mutant-type is negative predictor of Cmab-based therapy in mCRC. Previous studies have reported that the controlled disease rate of mCRC-treated Cmab-based therapy were 48 to 83% (mean, 67%) in patients with KRAS wild-type tumors and 10 to 74% (mean, 40%) in patients with KRAS mutant-type tumors (14,19,21,43,44). Our data also demonstrated that KRAS mutant-type tumors were correlated with a lower controlled disease rate.

The p53 tumor suppressor gene has been demonstrated to regulate cell cycle progression and apoptosis. p53 mutations are found in 40 to 60% of patients with colorectal cancer (33). Mutated p53 protein accumulates in the nucleus and is detected by IHC (33,45). This method has since been suggested to predict p53 mutations. A previous study has suggested that p53 mutations are predictive of Cmab sensitivity, particularly in patients without KRAS mutation (27). Therefore, we examined the correlation between p53 expression using IHC and the efficacy of Cmab-based therapy; however, no correlation was identified.

The Ki67 antigen recognizes the nuclei of proliferating cells throughout the cell cycle, except during the G0 and early G1 phases (33). The Ki67 labeling index is associated with tumor proliferation (46). Recent studies have reported that neoadjuvant chemoradiotherapy with Cmab decreased the levels of the Ki67 labeling index in rectal cancer (28). We hypothesized that the Ki67 labeling index reflects the efficacy of Cmab-based therapy in CRC; thus, we examined the correlation between Ki67 and the efficacy of Cmab-based therapy; however, no correlation was found.

According to univariate and multivariate analysis, the efficacy of Cmab was significantly associated with KRAS status and E-cadherin expression. Moreover, multivariate analysis also demonstrated that the two factors were independent predictors of the efficacy of Cmab-based therapy in mCRC.

In the KRAS wild-type tumors, E-cadherin-positive status was correlated with a higher controlled disease rate. When expression of E-cadherin was considered a positive predictor of Cmab in KRAS wild-type mCRC, both sensitivity and specificity were 87.5%. Moreover, in KRAS mutant-type tumors, all responders expressed E-cadherin. Our results suggest that the expression of E-cadherin may be a predictive marker of Cmab-based therapy in mCRC independently or in combination with KRAS status analysis. Since E-cadherin IHC is a comparatively simple method, it is easy to introduce as a biomarker of Cmab-based therapy. In addition, it is possible that the expression of E-cadherin may be predictive of sensitivity to panitumumab, a fully human monoclonal antibody targeting the EGFR. However, as our study had a small sample size and was retrospective, it is necessary to conduct a large, prospective clinical trial in order to confirm this finding. In conclusion, our results indicate that detection of the expression of E-cadherin via IHC may be a positive predictor of Cmab-based therapy in mCRC, and that the combination of E-cadherin IHC and KRAS analysis may be a more sensitive biomarker than KRAS analysis alone.

## Figures and Tables

**Table I:** Patient characteristics.

Characteristics	No.	%
No. of patients	36	
Age (years)		
Median	62.2	
Range	29–79	
Gender		
Male	24	67
Female	12	33
Site of tumor		
Colon	20	56
Rectum	16	44
Synchronous metastasis	23	66
Metachronous recurrence	22	63
Lines of treatment		
≤2	9	25
3	27	75
Concurrent chemotherapy		
Yes	18	50
No	18	50

**Table II:** KRAS mutation types.

	Types of mutations found in codon 12^a^						Codon 13^a^	Total
	
	Asp (GAT)	Val (GTT)	Ser (AGT)	Arg (CGT)	Cys (TGT)	Ala (GCT)	Asp (GTC)	
Number of tumors with each KRAS mutation/number of tumors with KRAS mutation (%)	5/12 (42)	4/12 (33)	1/12 (8)	0/12 (0)	0/12 (0)	0/12 (0)	2/12 (17)	12/36^a^

a Number of tumors with KRAS mutation/total number of tumors examined.

**Table III:** Response to treatment according to *KRAS* status and p53, Ki67 and E-cadherin IHC.

	Responder	Non-responder	P-value
KRAS status			
Wild-type	16	8	0.033^a^
Mutant	3	9	
p53 IHC			
Positive	17	12	0.219
Negative	2	5	
Ki67 IHC			
Positive	11	10	1.000
Negative	8	7	
E-cadherin IHC			
Positive	17	2	<0.001^a^
Negative	5	12	

a P<0.05. IHC, immunohistochemistry.

**Table IV:** Response to treatment according to combined *KRAS* status and E-cadherin IHC.

E-cadherin IHC	Responder	Non-responder	P-value
KRAS wild-type			
Positive	14	1	0.001^a^
Negative	2	7	
KRAS mutant type			
Positive	3	4	0.205
Negative	0	5	

a P<0.05. IHC, immunohistochemistry.

**Table V:** Univariate and multivariate analysis with respect to the efficacy of Cmab.

Variable	Univariate	Multivariate		
	
	P-value	Odds ratio	95% CI	P-value
KRAS status				
Wild-type vs. mutant	0.033	20.83	1.80–241.18	0.015^a^
E-cadherin				
Positive vs. negative	<0.001	54.91	4.53–664.89	0.002^a^

Cmab, cetuximab; CI, confidence interval.

a P<0.05.
